# Supplementary material for: Confidence control for efficient behaviour in dynamic environments
Source: Nat Commun. 2024 Oct 22;15:9089. doi: 10.1038/s41467-024-53312-3 (PMC11493976; doi:10.1038/s41467-024-53312-3)
Supplement: Supplementary file 1 — Supplementary Information [file 41467_2024_53312_MOESM1_ESM.pdf]

Supplementary Information for

Confidence control for efficient behaviour in dynamic environments

Tarryn Balsdon*[1,2] & Marios G Philiastides[1]

1. School of Psychology and Neuroscience, University of Glasgow, Glasgow, G12 8QB, United Kingdom
2. Laboratory of Perceptual Systems, DEC, ENS, PSL University, CNRS (UMR 8248), Paris, 75005, France

*Corresponding author: tarryn.balsdon@ens.fr

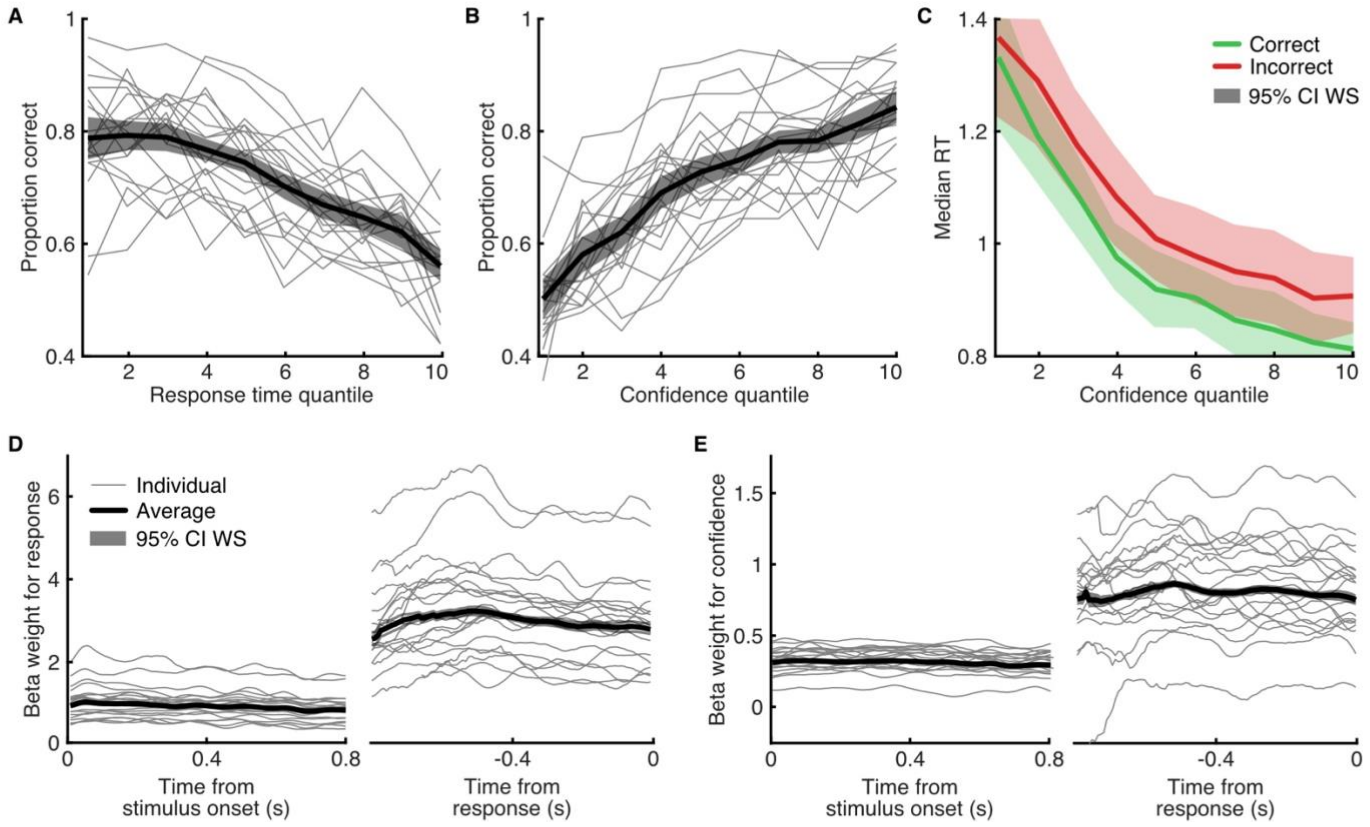

Supplementary Figure S1. Behavioural interactions. A) *Proportion correct by response time, where trials have been sorted into 10 quantiles of increasing response times within each participant (thin lines). The average is shown in the thick line, with 95% within-subjects confidence (across bins) in the shaded region.* ***B)*** *Proportion correct by confidence, sorting trials into 10 quantiles of increasing confidence. Same format as A).* ***C)*** *Median response time by confidence, for correct (light green) and error (dark red) trials separately, same quantiles as in B). Shaded region shows 95% within-subject confidence for the difference between correct and incorrect.* ***D)*** *Beta weights from a GLM analysis predicting responses from the presented stimulus evidence on each trial, locked to stimulus onset (left) and response time (right).* ***E)*** *Beta weight from a GLM analysis predicting confidence from the presented stimulus evidence on each trial, locked to stimulus onset (left) and response time (right). Thin lines show individual participants, thick line shows the average, and shaded region corresponds to 95% within-subjects confidence (across time, to highlight potential temporal differences).*

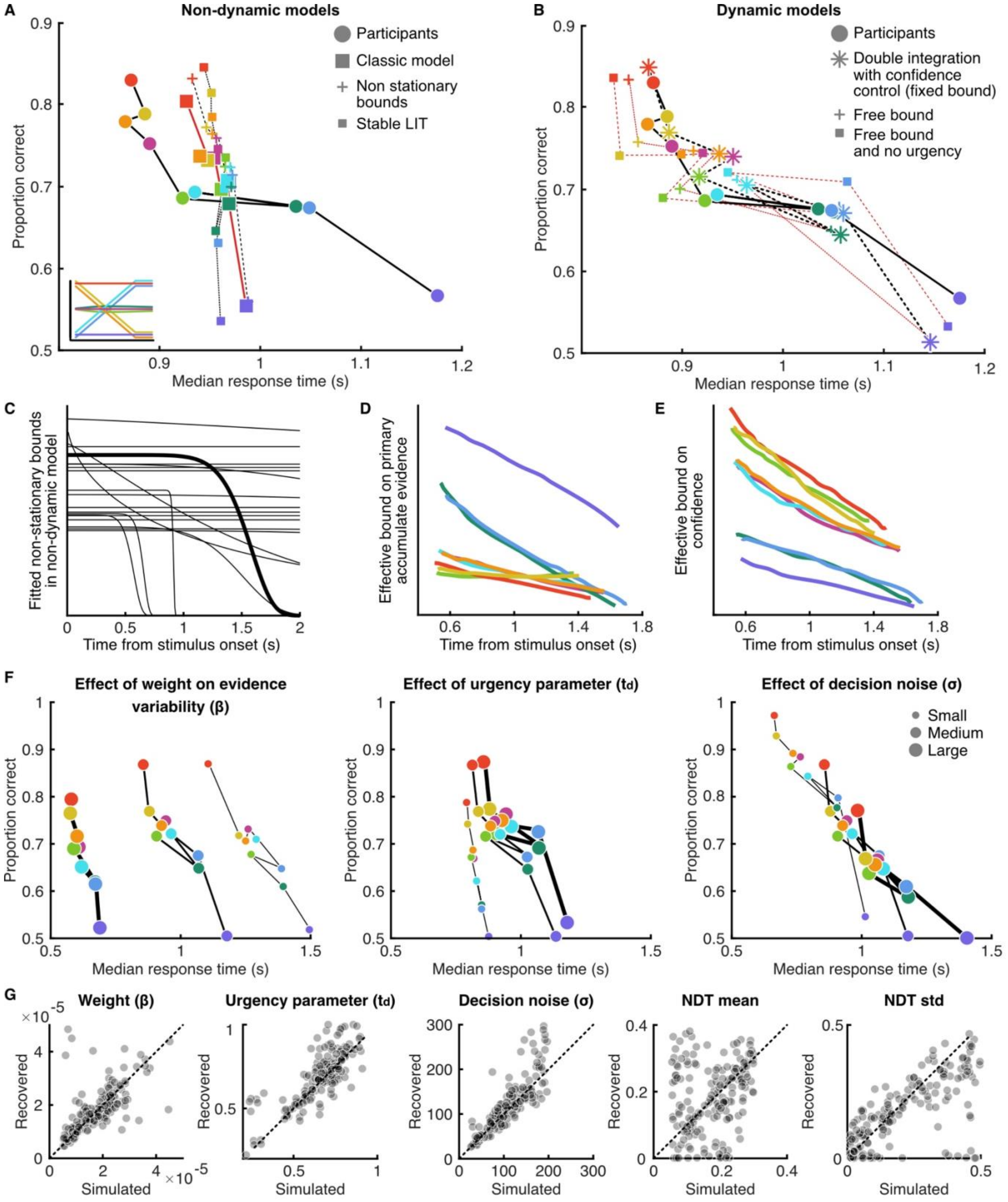

Supplementary Figure S2. Comparison of models and parameters. A) *Average participant (large circles) proportion correct by median response time across conditions, compared to non-dynamic model variants: large squares show the simple classic model presented in the main manuscript; crosses show this same model with non-stationary decision bounds (Weibull function); small squares show the stable leaky integrating threshold (LIT) model (original double-integration model; Verdonck et al., 2021), which takes the same double integration format as the dynamic models, except the motor integrator smoothing parameters is set to a fixed value, rather than being modulated by confidence. Colours show the conditions as in* ***Figure 1b*** *(inset).* ***B)*** *Average participant (large circles) behaviour compared to dynamic models. All models are variants of the double-integration model with confidence control. Asterisks show the final model included in the main manuscript, this is simplified compared to the model in crosses, which includes a motor bound parameter that is free to be*

adjusted across participants. Small squares show an initial model in which the motor bound parameter was also free, but did not include any control of urgency. This urgency control was added to the other two models, where motor smoothing decreased with increasingly lower confidence from a certain time-point, t_d. ***C)*** *Bounds for each participant from the parameters fit in the non-dynamic model with flexible, non-stationary bounds. The thick line shows the example participant in D) and E).* ***D)*** *Effective bounds on Primary accumulated evidence for one example participant, calculated by taking a running average primary evidence accumulated up to the time of decision commitment, separately for each condition. The figure suggests that if the participant were to implement behaviour suggested by the double-integration framework but with only a single-integration, bounds would need to not only differ across time, but with the evidence uncertainty.* ***E)*** *The same as in D) but for confidence, for comparison with models proposing a bound is set on confidence itself. Similar to Lee et al., 2023, the effective bounds are dependent on stimulus difficulty (condition) and decrease over time. Here this is mainly due to the combination of confidence smoothing and primary evidence reintegration at the motor level, as opposed to a prediction of the cost on continued accumulation.* ***F)*** *Demonstration of the effects of three critical model parameters on behaviour: the weight on evidence variability (β; left); the urgency parameter, marking time at which motor smoothing is influenced by lower confidence (t_d; middle); and decision noise. The small, medium, and large (thin to thick) lines correspond to the minimum, median, and maximum value of the parameters fit to participants, the other parameters were fixed to the median (see* ***Supplementary Table S4****). Both proportion correct and response time are influenced by all three parameters.* ***G)*** *Parameter recovery analysis for the parameters of the best-fitting model. Parameter values were sampled from a uniform distribution around the range of values fit to participant data. Behaviour in the experiment (with the same number of trials) was simulated using the model, and then the model was fit to this simulated behaviour to obtain the recovered parameters. The mean and standard deviation of the non-decision time (NDT, rightmost plots) were sometimes confused (a larger mean substituted for a smaller variance), but the recovery of the behaviourally relevant process parameters (weight, urgency, and decision noise) was acceptable.*

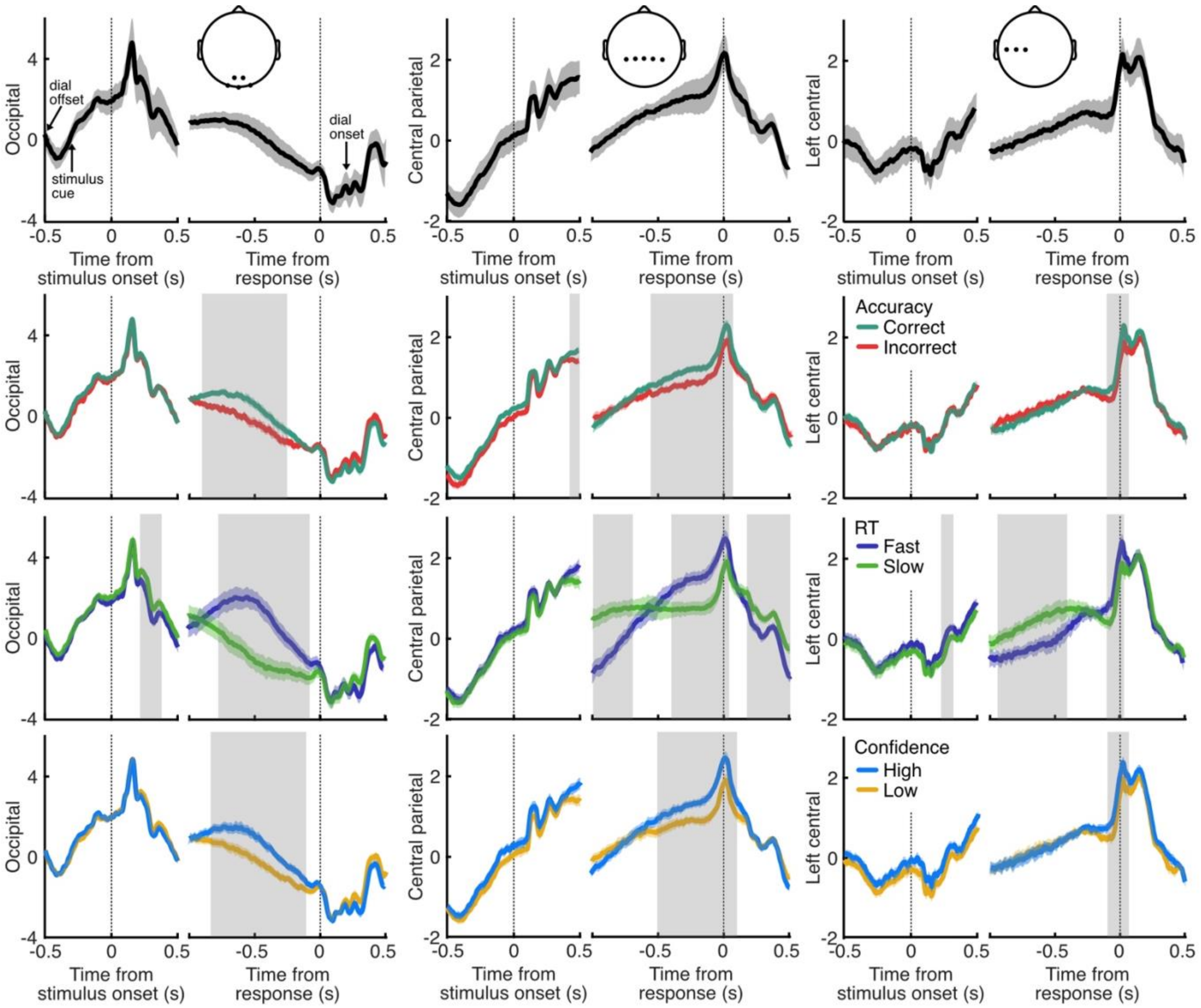

Supplementary Figure S3. Traditional EEG analyses. *From left to right, traces show average of z-scored occipital, central parietal, and left central electrodes (inserts of topography). The grand average is shown at the top, with trials split by accuracy, response time, and confidence (respectively) below. Shaded error bars correspond to 95% within-subject confidence (difference between conditions; or between-subject confidence for the grand average). Vertical grey shaded regions mark time periods of cluster-corrected significance.*

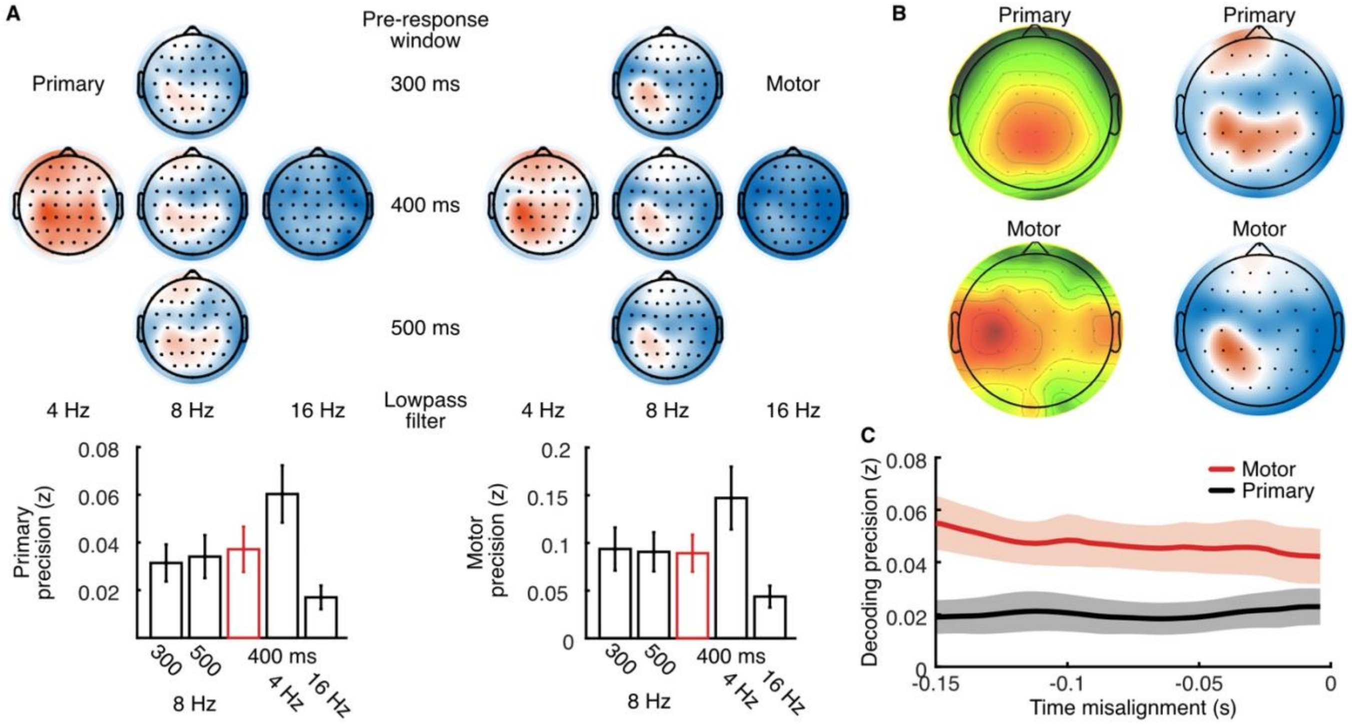

Supplementary Figure S4. Choices for EEG decoding analyses. A) *Topographies for primary (left) and motor (right) accumulators by choice of lowpass filter (horizontal) and pre-response time-window (vertical), with the central filter/window that shown in the main manuscript. While decreasing the lowpass filter improves performance, it also decreases specificity. The choice of window had less effect, but reflects a balance of including more time to better capture the primary weights while also reducing the time-window to minimise the correlation between model predicted primary and motor accumulator traces, and biases where less time can be included from trials with faster responses. (Below) Precision of the decoding of primary (left) and motor (right) accumulators based on filter and window choices. Precision is calculated as the correlation between actual and EEG predicted value, using 10-fold cross-validation. Error bars show 95% within-subject confidence intervals (although precision is low it is significantly above theoretical chance, 0). The red bar marks that included in the manuscript. All analyses use separate encoding models for primary and motor* ***B)*** *Topography from Balsdon et al., 2023, where a different analysis pipeline was used (left). Topography resulting from the decoding analysis performed separately for primary and motor accumulators (right). In the manuscript we present the topographies when the analysis is performed for both accumulators simultaneously, where the weights become parcellated between the two accumulators.* ***C)*** *Decoding precision (10-fold cross validation) for motor and primary accumulators, performing the analysis with the computational model estimates pulled backward in time (using simultaneous analysis, multiple linear inverse encoding model). We had assumed we might see increased decoding performance aligning the model estimates ~50 ms prior to the response, to allow time for the cortical signal to reach the finger. This was not the case, perhaps due to variability in the real alignment. For all topographies, more red indicates stronger decoding weight, in units normalised across analyses (except for the previous results in B).*

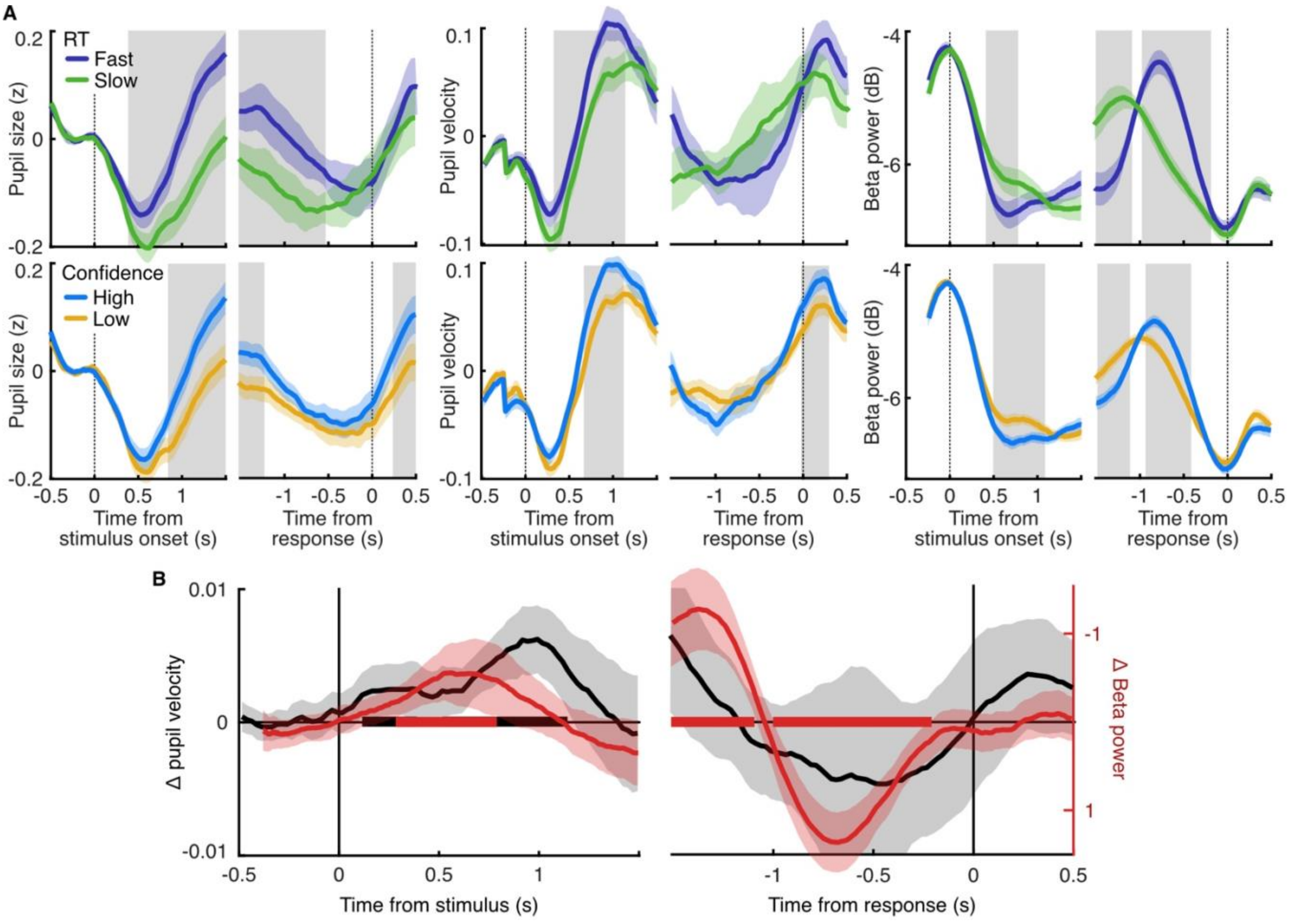

***Supplementary Figure S5. Decision preparation signals**. **A)** Effect of response speed (top row) and confidence (bottom row) on pupil size, pupil derivative, and motor beta power. In all cases a median split was used, within participants. Shaded error shows 95% within-subjects confidence (difference between split). Grey shaded regions show significant differences at the group level. Motor beta power was computed from the power spectrum across frequency tapers from 16 to 32 Hz with 25% spectral smoothing, resolved using wavelet convolution in FieldTrip. Channels were selected for each participant as the channel with the greatest (negative slope on beta power in the response epoch (C3 for 8 participants, and FC1, C1, and FC3 for 5, 4, and 3 participants respectively). **B)** Difference in the pupil derivative (velocity of change in pupil size; black) and beta power (red) using a median split of response times (fast – slow RTs). To make beta power better comparable with pupil size, the y-axis is reversed, such that decreased beta power corresponds to increased pupil size, the direction thought to indicate response preparation. Shaded regions show the 95% between-subjects confidence intervals. Thick lines at y = 0 mark regions of cluster-corrected significance for the difference in pupil velocity (black) and beta power (red) based on the median split of response times. Fast responses were associated with faster pupil dilation following stimulus onset (cluster corrected significant window, 0.125 to 1.15 s; mean t(18) = 3.28, mean p = 0.018). The same median split of response times showed an appreciably similar effect on motor Beta power (red line; cluster corrected significant window 0.28 to 0.79 s; mean t(18) = 2.75, mean p = 0.018; the effects in the response-locked epoch also follow a similar shape, but the pupil derivative is too variable to reach significance). While preliminary in nature, this finding is in line with the hypothesis that motor leakage is implemented via subcortical inhibition, reflected in part in pupil dilation and resulting in decreased cortical motor Beta power with the release from inhibition.*

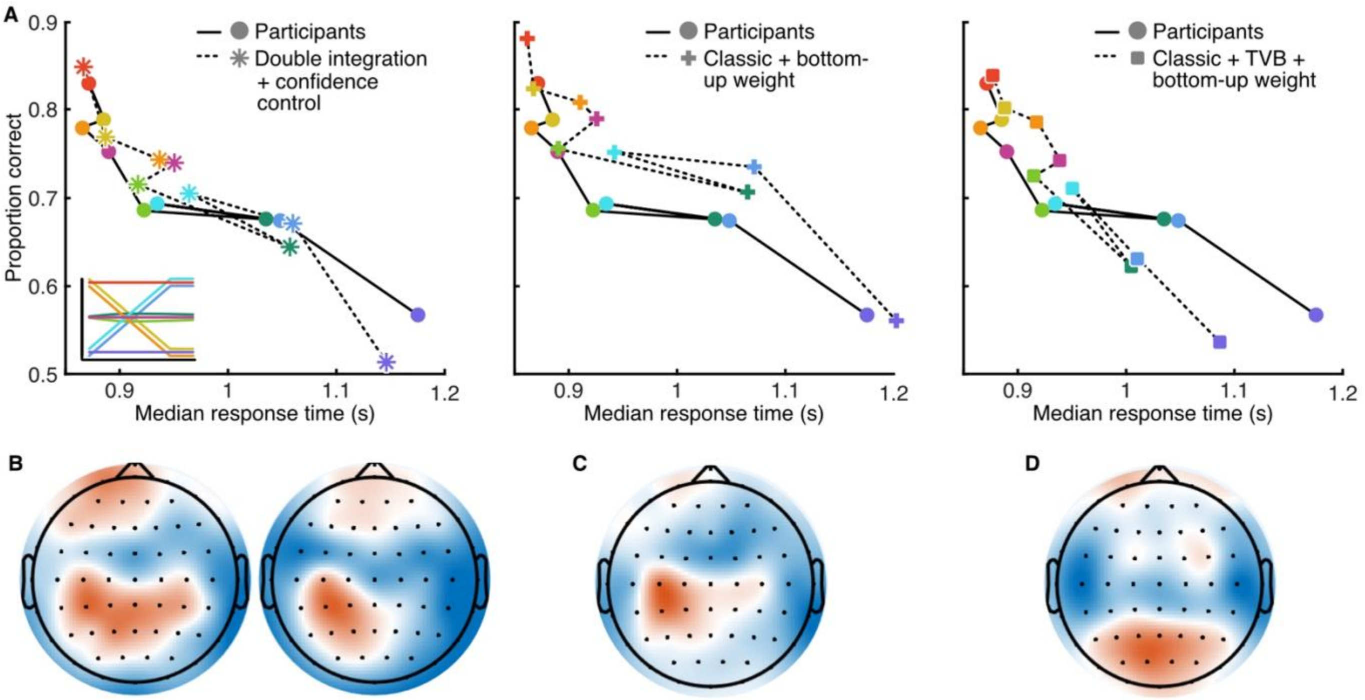

Supplementary Figure S6. Examining possible bottom-up mechanisms*. The double-integration model with confidence control (left panel of* ***A****) used an estimate of the variability of the decision evidence in computing confidence, which then affects the leak in the motor accumulator. We examined whether a weighting on the variability of the decision evidence could explain behaviour in a bottom-up manner, that is, affecting the primary evidence as it is accumulated in a single-integration framework, as presented in Figure 3b in the main manuscript. Note that the variability of the stimulus evidence is already taken account of in the decision evidence (Equations 1 – 3 in the manuscript), so this would amount to an over/under-weighting of the variability at the decision level. Using an additional free parameter, we fit the classic single-integration model with a weight on the decision evidence variability as in Equation 11 of the manuscript. This did improve the fit to behaviour (middle panel of* ***A****) relative to the other single-integration models, but BIC was larger (*$\sum BIC = 83\,385$*, see Supplementary Table S4) due to the extra parameter. Overall, the model overestimated performance while showing relatively improved estimates of response times. Allowing non-linear bounds in addition (right panel of* ***A****) also showed some improvement, but again the additional parameters did not make the fit parsimonious (*$\sum BIC = 83\,403$*). Neither model was found to be superior to the double-integration model with confidence control. Still, we questioned whether the latent model processes could possibly provide a better description of the EEG, given they rely on the single-integration process traditionally associated with central-parietal positivity. On the contrary, in order to fit behaviour, the classic model with the bottom-up weight estimated evidence accumulation more similar to the motor accumulator of the double-integration model, both numerically (average correlation in accumulator traces, r = 0.9) and in terms of the topography of the associated EEG signatures (****B****, left, vs* ***C****; though these signatures were less predictive of the latent processes, z = 0.044, compared to z = 0.091 for the motor accumulator in the double-integration model). The latent processes of the classic model with time-varying bounds and with bottom-up weight were localised to occipital regions (****D****), which is incongruous with the literature on neural signatures of evidence accumulation (and also not so well predicted, z = 0.058). One could also consider that even if the bottom-up models did produce EEG predictions that made theoretical sense, it would be difficult to compete with the double-integration model in capturing EEG signatures across the full extent of the two accumulators (both central-parietal and motor signatures). Further, we showed in the manuscript that the double-integration model also provides a good prediction of post-decision confidence, whereas single-integration models require additional mechanisms to predict confidence (such as ongoing accumulation to a secondary bound). While bottom-up explanations may be appealing in terms of their mechanistic simplicity, the double-integration framework could be considered more comprehensive in terms of the additional behaviour and neural processes it encompasses. For all topographies, more red indicates stronger decoding weight, in units normalised across analyses.*

p → t(18) ↓	1.87E-03	3.47E-03	3.06E-05	7.80E-08	6.24E-10	2.67E-07	1.35E-07	2.63E-11
3.555		0.783	0.024	8.24E-07	6.42E-06	4.02E-06	1.47E-06	1.43E-10
3.292	0.280		0.006	4.44E-07	2.87E-06	2.57E-05	7.37E-07	2.19E-09
5.286	2.424	3.080		1.39E-04	8.20E-05	7.50E-04	6.34E-06	9.43E-09
8.027	6.892	7.182	4.645		0.829	0.313	0.612	8.07E-07
10.662	5.964	6.321	4.868	0.218		0.325	0.925	4.94E-06
7.423	6.171	5.360	3.940	-1.034	-1.008		0.107	4.85E-08
7.756	6.625	6.944	5.969	0.515	0.095	1.683		2.43E-06
12.677	11.569	9.930	9.123	6.901	6.080	8.266	6.398	↑ p ← t(18)

Supplementary Table S1. Pairwise t-test statistics, effect of stimulus condition on discrimination performance (d'). *Left of the diagonal provides the t-statistics, right provides p-values (two-tailed), with values less than the Bonferroni corrected significance level ($p < 0.0014$) highlighted in red.*

	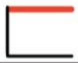	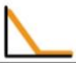	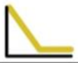	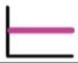	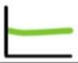	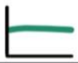	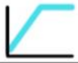	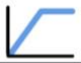	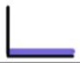
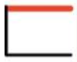	p → t(18)↓	0.253	0.751	0.135	4.11E-03	2.84E-05	2.85E-03	5.41E-06	1.44E-06
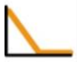	-1.175		0.141	0.735	3.70E-02	1.13E-04	1.89E-02	2.19E-05	3.29E-06
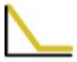	0.322	1.528		0.041	2.83E-03	1.44E-05	5.73E-04	5.36E-06	1.13E-06
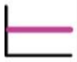	-1.556	-0.344	-2.175		0.075	3.72E-05	4.51E-03	1.10E-05	2.91E-06
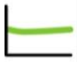	-3.219	-2.228	-3.380	-1.876		3.49E-04	0.615	1.06E-04	2.36E-06
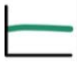	-5.318	-4.731	-5.610	-5.203	-4.260		1.18E-04	0.950	3.32E-05
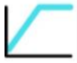	-3.377	-2.543	-4.053	-3.180	-0.511	4.716		4.09E-05	2.43E-06
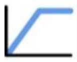	-6.039	-5.429	-6.043	-5.728	-4.759	0.064	-5.162		5.23E-05
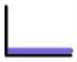	-6.634	-6.261	-6.744	-6.315	-6.410	-5.251	-6.396	-5.058	↑p ←t(18)

Supplementary Table S2. Pairwise t-test statistics, effect of stimulus condition on response time (log(RT)). *Left of the diagonal provides the t-statistics, right provides p-values (two-tailed), with values less than the Bonferroni corrected significance level ($p < 0.0014$) highlighted in red.*

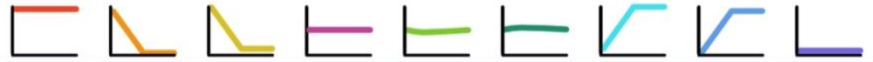

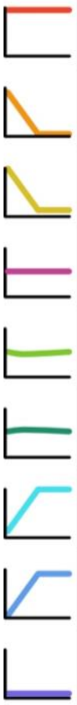

	p → t(18) ↓	0.001	0.047	0.083	2.95E-04	8.89E-08	9.24E-03	1.46E-04	6.23E-07
	3.898		0.108	0.226	0.042	9.68E-05	0.598	0.014	2.10E-06
	2.112	-1.679		0.948	0.010	2.30E-06	0.027	9.32E-04	1.06E-06
	1.823	-1.248	0.065		0.002	7.82E-06	0.095	3.58E-04	1.14E-06
	4.331	2.161	2.829	3.458		9.00E-03	0.225	0.092	2.53E-06
	7.962	4.798	6.422	5.877	2.878		1.40E-04	0.186	9.00E-06
	2.866	0.535	2.370	1.746	-1.250	-4.642		3.91E-03	2.03E-06
	4.626	2.693	3.849	4.249	1.765	-1.366	3.242		6.10E-06
	7.022	6.463	6.776	6.742	6.379	5.815	6.477	5.986	↑p ←t(18)

Supplementary Table S3. Pairwise t-test statistics, effect of stimulus condition on median confidence.
Left of the diagonal provides the t-statistics, right provides p-values (two-tailed), with values less than the Bonferroni corrected significance level ($p < 0.0014$) highlighted in red.

	**b**	$\boldsymbol{\sigma}$	μ_U	σ_U	$\boldsymbol{\lambda}$	$\boldsymbol{\beta}$	$\boldsymbol{t_d}$	**NLL**	**ΣBIC**	**Protected exceedance probability**		
**Classic**	82.82	3.29	0.18	0.20				2070.15	83346.78	0.094	0.995	
**Classic TVB**	74.53	2.65	0.23	0.34				2068.26	83637.23	0.028	0.000	
**LIT**	1.98E-03	17.67	0.27	0.43	9.64E-05			2073.01	83596.51	0.028	0.004	
**free bound**	1.76E-02	12.13	0.22	0.05		2.90E-05		2049.84	82669.37	0.190		0.379
**add urgency**	3.19E-02	11.51	0.23	0.14		3.14E-05	1.39	2044.97	82610.05	0.329		0.310
**fixed bound**		10.99	0.21	0.20		2.59E-05	1.34	2045.71	82504.17	0.331		0.310

Supplementary Table S4. Average parameters and model fit statistics. *Classic TVB refers to the version with time-varying bounds (the parameters for the time-varying component aren't shown, but the bounds themselves are plotted in* ***Supplementary Figure 2C****). The LIT refers to the Leaky Integration Threshold model, the original double-integration model with leakage stable over time. The three models with confidence control are below, first with a free bound on the motor accumulator, then adding urgency via increased leakage, and finally fixing the bound. In terms of protected exceedance probability, these dynamic models were not differentiable, the manuscript presents the fixed bound model, which has the smallest sum of BIC.*
