# Supplementary material for: Confidence control for efficient behaviour in dynamic environments
Source: Nat Commun. 2024 Oct 22;15:9089. doi: 10.1038/s41467-024-53312-3 (PMC11493976; doi:10.1038/s41467-024-53312-3)
Supplement: Supplementary file 3 — Reporting Summary [file 41467_2024_53312_MOESM3_ESM.pdf]

nature portfolio

Corresponding author(s): Tarryn Balsdon

Last updated by author(s): 11 September 2024

Reporting Summary

Nature Portfolio wishes to improve the reproducibility of the work that we publish. This form provides structure for consistency and transparency in reporting. For further information on Nature Portfolio policies, see our Editorial Policies and the Editorial Policy Checklist.

Statistics

For all statistical analyses, confirm that the following items are present in the figure legend, table legend, main text, or Methods section.

n/a	Confirmed	
☐	☒	The exact sample size (*n*) for each experimental group/condition, given as a discrete number and unit of measurement
☐	☒	A statement on whether measurements were taken from distinct samples or whether the same sample was measured repeatedly
☐	☒	The statistical test(s) used AND whether they are one- or two-sided<br>*Only common tests should be described solely by name; describe more complex techniques in the Methods section.*
☐	☒	A description of all covariates tested
☐	☒	A description of any assumptions or corrections, such as tests of normality and adjustment for multiple comparisons
☐	☒	A full description of the statistical parameters including central tendency (e.g. means) or other basic estimates (e.g. regression coefficient) AND variation (e.g. standard deviation) or associated estimates of uncertainty (e.g. confidence intervals)
☐	☒	For null hypothesis testing, the test statistic (e.g. *F*, *t*, *r*) with confidence intervals, effect sizes, degrees of freedom and *P* value noted<br>*Give P values as exact values whenever suitable.*
☒	☐	For Bayesian analysis, information on the choice of priors and Markov chain Monte Carlo settings
☒	☐	For hierarchical and complex designs, identification of the appropriate level for tests and full reporting of outcomes
☒	☐	Estimates of effect sizes (e.g. Cohen's *d*, Pearson's *r*), indicating how they were calculated

Our web collection on statistics for biologists contains articles on many of the points above.

Software and code

Policy information about availability of computer code

Data collection	Stimulus presentation was controlled using custom code in Matlab (version 2022a) and the Psychophysics toolbox. Gaze and pupil dilation were monitored using video-based pupil and corneal reflection, controlled using the Titta toolbox for Matlab.
Data analysis	Data analysis was conducted using mainly custom code in Matlab (version 2022a), available on the open science framework (https://osf.io/gky5a/) alongside the pre-registration and data. EEG pre-processing was performed using EEGLAB (version 14.1.2b). Additional toolboxes: Fieldtrip plug-in for EEGLAB (version 161018), prepPipeline plug-in fro EEGLAB (version 0.55.3), with ICLabel (version 0.3). Model fitting used the BADS algorythm (version 1.0.5) and model comparison used the BMS function from SPM (version 12) for MATLAB.

For manuscripts utilizing custom algorithms or software that are central to the research but not yet described in published literature, software must be made available to editors and reviewers. We strongly encourage code deposition in a community repository (e.g. GitHub). See the Nature Portfolio guidelines for submitting code & software for further information.

Data

Policy information about availability of data

All manuscripts must include a data availability statement. This statement should provide the following information, where applicable:

- Accession codes, unique identifiers, or web links for publicly available datasets
- A description of any restrictions on data availability
- For clinical datasets or third party data, please ensure that the statement adheres to our policy

Data are available on the open science framework: https://osf.io/d7ea8/
This includes a csv summary of behavioural data, behavioural data in MATLAB, raw EEG data , and raw pupillometry

Research involving human participants, their data, or biological material

Policy information about studies with human participants or human data. See also policy information about sex, gender (identity/presentation), and sexual orientation and race, ethnicity and racism.

Reporting on sex and gender	We do not perform analyses based on sex, gender, nor sexual orientation. We had no theoretically motivated hypothesis as to why we may or may not see a difference based on these variables. We did not obtain ethical approval from participants to share data relating to their sex nor gender
Reporting on race, ethnicity, or other socially relevant groupings	We did not collect data relating race, ethnicity or other socially relevant grouping.
Population characteristics	Participants were recruited from the Experimental Subject Pool in the School of Psychology (University of Glasgow), who indicated the absence of diagnosed mental disorders, and had normal or corrected-to-normal vision. The subject pool contains both students and non-students, all participants are over 18.
Recruitment	Participants were recruited from the Experimental Subject Pool in the School of Psychology (University of Glasgow), who indicated the absence of diagnosed mental disorders, and had normal or corrected-to-normal vision. Participants were recruited in order of response to the advertisement. Self-selection biases may include: an interest in psychology or cognitive science, work restrictions related to UK student visas. These are unlikely to influence the results.
Ethics oversight	Ethical approval for this study was granted by the College of Science and Engineering Ethics Committee at the University of Glasgow (application number 200210194).

Note that full information on the approval of the study protocol must also be provided in the manuscript.

Field-specific reporting

Please select the one below that is the best fit for your research. If you are not sure, read the appropriate sections before making your selection.

☒ Life sciences ☐ Behavioural & social sciences ☐ Ecological, evolutionary & environmental sciences

For a reference copy of the document with all sections, see nature.com/documents/nr-reporting-summary-flat.pdf

Life sciences study design

All studies must disclose on these points even when the disclosure is negative.

Sample size	We aimed to include 20 participants, replacing participants whose performance did not rise significantly above chance (58.3% correct, given 100 trials per condition, 2 excluded), whose reaction times were too slow (median > 2 s, the maximum duration of the stimulus, none excluded), or with poor quality EEG (3 excluded, for technical issues).<br>Sample size determination: With 100 trials per condition, we can detect a significant difference of 8.5% from 75% correct within individual participants. We expected larger differences than this across stimulus conditions (ranging from 60 to 90 % correct). A sample size of 20 participants was chosen to demonstrate the generalisability of the within-subject findings, and for the purpose of the EEG and pupillometry analyses.
Data exclusions	We excluded two participants for poor task performance (less than 58.3% correct: greater than 58.3% correct is significantly above chance given 100 trials per condition), and three participants for technical issues with the EEG (battery failure).
Replication	We have not performed a replication experiment.
Randomization	All analyses were within-subjects, there was no grouping. Trials from different experimental conditions were randomly intermixed.
Blinding	There was no blinding (all participants completed all conditions).

Reporting for specific materials, systems and methods

We require information from authors about some types of materials, experimental systems and methods used in many studies. Here, indicate whether each material, system or method listed is relevant to your study. If you are not sure if a list item applies to your research, read the appropriate section before selecting a response.

Materials & experimental systems

n/a	Involved in the study
☒	☐ Antibodies
☒	☐ Eukaryotic cell lines
☒	☐ Palaeontology and archaeology
☒	☐ Animals and other organisms
☒	☐ Clinical data
☒	☐ Dual use research of concern
☒	☐ Plants

Methods

n/a	Involved in the study
☒	☐ ChIP-seq
☒	☐ Flow cytometry
☒	☐ MRI-based neuroimaging

Plants

Seed stocks	*Report on the source of all seed stocks or other plant material used. If applicable, state the seed stock centre and catalogue number. If plant specimens were collected from the field, describe the collection location, date and sampling procedures.*
Novel plant genotypes	*Describe the methods by which all novel plant genotypes were produced. This includes those generated by transgenic approaches, gene editing, chemical/radiation-based mutagenesis and hybridization. For transgenic lines, describe the transformation method, the number of independent lines analyzed and the generation upon which experiments were performed. For gene-edited lines, describe the editor used, the endogenous sequence targeted for editing, the targeting guide RNA sequence (if applicable) and how the editor was applied.*
Authentication	*Describe any authentication procedures for each seed stock used or novel genotype generated. Describe any experiments used to assess the effect of a mutation and, where applicable, how potential secondary effects (e.g. second site T-DNA insertions, mosiacism, off-target gene editing) were examined.*
